# 

**Published:** 1892-04

**Authors:** 


### CONTENTS:

Arricle I.—Concentration as a Principle of Success Or, "One Thing I Do." By Rev. N. T. Roberts. - - - 529
" II.—Post-Graduate Study. By R. B. Tuller. - - 541
" III.—The Didactic Method in Dental Teaching. By C. M. Wright, D. D. S. - - - - - - - 548
" IV.—Diseases of the Oral Mucous Membrane. By J. D. Patterson. - - - - - - - - - 553
" V.—How Gas Cylinders are Made. - - - - 556
" VI.—Treatment and Destruction of the Pulp. By Dr. Frank French. - - - - - - - - 560
" VII.—Notes on a Case of Fracture of the Superior Maxillæ. 564

### EDITORIAL.

Dentists and the Census Bill, - - - - - - - 566
The Texas Dental Law, - - - - - - - - 568
Baltimore College of Dental Surgery, - - - - - - 570
University of Maryland—Dental Department, - - - 572
Cincinnati College of Medicine and Surgery—Dental Department, 573

### COMMUNICATIONS.

American Academy of Dental Science, - - - - - - 573

American Dental Society of Europe, - - - - - - 574

Kentucky State Dental Association, - - - - - - 575

National Association of Dental Examiners, - - - - 575

### MONTHLY SUMMARY.

To Students, - - - - - - - - - - - 575
Acidity of the Stomach, - - - - - - - - 576

